# Non-fungible tokens in healthcare: a scoping review

**DOI:** 10.3389/fpubh.2023.1266385

**Published:** 2023-11-21

**Authors:** Shahabeddin Abhari, Plinio Morita, Pedro Augusto Da Silva E. Souza Miranda, Ali Garavand, Thokozani Hanjahanja-Phiri, Dmytro Chumachenko

**Affiliations:** ^1^School of Public Health Sciences, University of Waterloo, Waterloo, ON, Canada; ^2^Department of Systems Design Engineering, University of Waterloo, Waterloo, ON, Canada; ^3^Research Institute for Aging, University of Waterloo, Waterloo, ON, Canada; ^4^Centre for Digital Therapeutics, Techna Institute, University Health Network, Toronto, ON, Canada; ^5^Dalla Lana School of Public Health, Institute of Health Policy, Management, and Evaluation, University of Toronto, Toronto, ON, Canada; ^6^Department of Health Information Technology, School of Allied Medical Sciences, Lorestan University of Medical Sciences, Khorramabad, Iran; ^7^Department of Mathematical Modelling and Artificial Intelligence, National Aerospace University “Kharkiv Aviation Institute”, Kharkiv, Ukraine

**Keywords:** non-fungible tokens, blockchains, healthcare, metaverse, medical, NFT

## Abstract

**Introduction:**

Non-Fungible Tokens (NFTs) are digital assets that are verified using blockchain technology to ensure authenticity and ownership. NFTs have the potential to revolutionize healthcare by addressing various issues in the industry.

**Method:**

The goal of this study was to identify the applications of NFTs in healthcare. Our scoping review was conducted in 2023. We searched the Scopus, IEEE, PubMed, Web of Science, Science Direct, and Cochrane scientific databases using related keywords. The article selection process was based on Preferred Reporting Items for Systematic Reviews and Meta-Analyses (PRISMA).

**Results:**

After applying inclusion and exclusion criteria, a total of 13 articles were chosen. Then extracted data was summarized and reported. The most common application of NFTs in healthcare was found to be in health data management with 46% frequency, followed by supply chain management with 31% frequency. Furthermore, Ethereum is the main blockchain platform that is applied in NFTs in healthcare with 70%.

**Discussion:**

The findings from this review indicate that the NFTs that are currently used in healthcare could transform it. Also, it appears that researchers have not yet investigated the numerous potentials uses of NFTs in the healthcare field, which could be utilized in the future.

## 1 Introduction

With the pressure of long-term chronic disease, rising costs of services, aging population, an inadequate health workforce, and finite resources, we have long known that the present healthcare system is unsustainable. With the recent growth of digital technologies, the healthcare industry is poised to shift toward digital health as a means of addressing the various challenges it faces (1–4). Digital Health is not only transforming the provision of care directly but is also becoming a crucial facilitator of change in the health system. The COVID-19 pandemic has hastened and assisted this paradigm shift away from traditional healthcare toward digital health (5–8). Also, as the age of the Metaverse rapidly approaches, there is growing evidence that the metaverse can be applied to healthcare. For instance, telemedicine, medical education, mental health therapy, patient education, and medical research are some applications of the Metaverse (9, 10).

For several years running, Gartner a technological research and consulting firm has ranked blockchain as one of the top 10 strategic technology trends (9, 11–14). This technology is predicted to transform numerous businesses and usher in new revenue streams in a variety of areas, including the healthcare (14–18). Digital assets, which are foundational elements in the realm of blockchain technology, encompass an array of assets, primarily manifesting as cryptocurrencies and tokens. These digital assets serve distinct purposes within the blockchain ecosystem. Tokens, for instance, fulfill various roles in this landscape, representing either fungible or non-fungible assets. The dichotomy of tokens gives rise to two broad categories: fungible tokens and non-fungible tokens (NFTs). Both cryptocurrencies, exemplified by Bitcoin, and NFTs are digital assets verified and secured through blockchain technology. Cryptocurrencies, exemplified by Bitcoin, fall into the category of fungible tokens, serving as a standardized unit of value exchange within the digital landscape. They are chiefly utilized as mediums of trade or stores of value and are interchangeable with one another. Conversely, NFTs, standing for non-fungible tokens, represent a distinct category of digital assets designed to signify ownership or establish provenance for unique digital assets. In essence, NFTs function as digital certificates of authenticity, ensuring the uniqueness and origin of the underlying asset. Unlike cryptocurrencies, NFTs are not interchangeable due to their uniqueness, and each one represents a one-of-a-kind digital item. Cryptocurrencies derive their value from market demand and are tokens representing specific units of currency, whereas NFTs derive their worth from the distinctiveness of the digital asset they validate. Both NFTs and cryptocurrencies are transferable digital assets, albeit NFT transfers may be subject to specific limitations established by the NFT's creator or owner (19–28).

According to Peres et al. (29), NFTs are “cryptographic assets on a blockchain that contain unique identifying information and codes that separate them from one another.” NFTs are frequently employed in digital art ownership, gaming assets, collectibles, real estate, identity verification, charitable donations and more (30–33). There are numerous examples of NFTs applications in the commercial and economic sphere (34) including in the art domain (35) and the gaming industry (36). Based on other investigations, NFTs have a wide range of applications across various industries, such as art (37), music (38), sports (39), gaming (40), and real estate (41). In the art world, NFTs have enabled artists to sell their digital art as unique, one-of-a-kind pieces, rather than selling multiple copies. It should be noted artists also can sell multiple copies of the same piece of art, as multiple NFTs, though (35, 37). In the music industry, NFTs can be used to authenticate and sell limited edition or exclusive music content (38). In sports, NFTs can be used to create unique collectibles, such as virtual trading cards or game highlights (39). In gaming, NFTs can be used to create rare in-game items or characters that can be traded or sold among players (12, 40). And in real estate, NFTs serve two main purposes. First, they act as digital proof of property ownership, providing secure records on the blockchain. Second, NFTs enable fractional ownership, dividing real estate assets into tradable units, making real estate investment more accessible and diverse (41).

## 2 Background

Bao and Roubaud (22) conducted a thorough evaluation of NFTs-related studies in 2023. The 13 articles included in this study were evaluated. Their findings revealed that all studies published in economics and finance journals after 2021, were primarily empirical and focused on the topic of asset pricing. Also, Taherdoost (28) conducted a second systematic evaluation of 34 studies in 2023 on NFTs-related research. According to the study's findings, the majority of articles on NFTs were published after 2020 and were mainly from the fields of engineering, computer science, economics, and material science.

**Figure 1:**
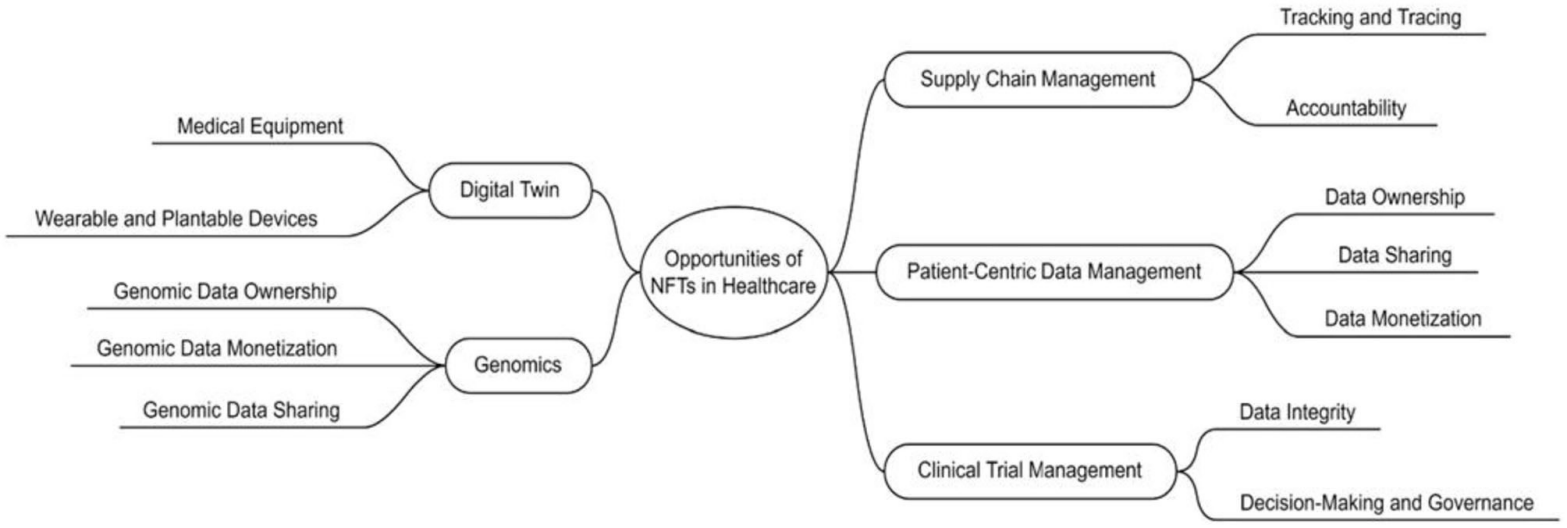
Opportunities of NFTs in healthcare based on Musamih et al. (44).

Although there is much evidence for the use of NFTs in the cited fields, there has not been much study on its use in healthcare. Recently, three major studies on the value of NFTs and its part in the future of healthcare were published. In the first recent study published in Nature Medicine, researchers mentioned that NFTs in healthcare might revolutionize how health data is handled. They discussed transparency, accountability, privacy, and patient ownership as being central to NFTs, which also important ideals in the field of healthcare. The authors of the study concluded that if NFTs are to revolutionize digital health, more improvements to protect data security, streamline operations, and promoting accessibility for all will be necessary (42). Another study conducted in 2022, highlighted the role and significance of NFTs in healthcare and focused on their various applications in the field. After introducing the concept of NFTs and their potential benefits, the study delved into specific use cases in healthcare such as simplifying blood and stem cell production and supply chain management (43). In a different insightful study, researchers spoke about the potential benefits that NFTs might have in terms of supply chain management, patient-centric data management, digital twins for research, clinical trial management, and genomics, among other healthcare-related fields. They explored several significant issues that are impeding the use of NFTs in the healthcare industry and offered suggestions for future research topics. Figure 1 shows how these NFTs opportunities can be in healthcare (44).

Despite the growing interest in exploring the potential applications of NFTs in healthcare (44, 45), there is a lack of systematic research on this topic. This scoping review aims to fill this gap by providing a comprehensive analysis of the current state of knowledge on the use of NFTs in healthcare. Now due to the absence of a thorough evaluation in this sector, the purpose of this study is to undertake a systematic search of the applications of NFTs in healthcare. This is the first scoping review study that was performed for applications of NFTs in healthcare. By providing a comprehensive overview of the current state of knowledge on the use of NFTs in healthcare, this scoping review aims to contribute to the development of a deeper understanding of the potential benefits of this emerging technology. This review will be of interest to healthcare providers, policymakers, and researchers who are interested in exploring the potential applications of NFTs in healthcare, and to those who wish to develop strategies for improving the efficiency, transparency, and security of healthcare delivery.

## 3 Methods

The primary objective of this investigation was to delineate the applications of NFTs within the domain of healthcare. In the year 2023, we executed a comprehensive scoping review, meticulously querying prestigious scientific databases, namely Scopus, IEEE, PubMed, Web of Science, Science Direct, and Cochrane. Our search was guided by relevant keywords, and the selection of articles adhered to the guidelines outlined in the Preferred Reporting Items for Systematic Reviews and Meta-Analyses (PRISMA). Following the application of predefined inclusion and exclusion criteria, a meticulous curation process led to the inclusion of a total of 13 articles. Subsequently, the collated data underwent meticulous summarization and reporting. Further elucidation of the methodological aspects is presented in given below.

### 3.1 Research question

RQ1: What are the main applications of NFTs in healthcare?

RQ2: What is the current state of NFTs usage in healthcare?

### 3.2 Information sources

The search started in February and ended on the first of March 2023. Searches were done in scientific databases, including Scopus, PubMed, Web of Science, IEEE, Science Direct, and Cochrane. Given the interdisciplinary nature of our research, we extended our search to encompass databases and knowledge bases in the fields of health sciences, engineering, and management.

### 3.3 Search strategy

**Figure 2:**
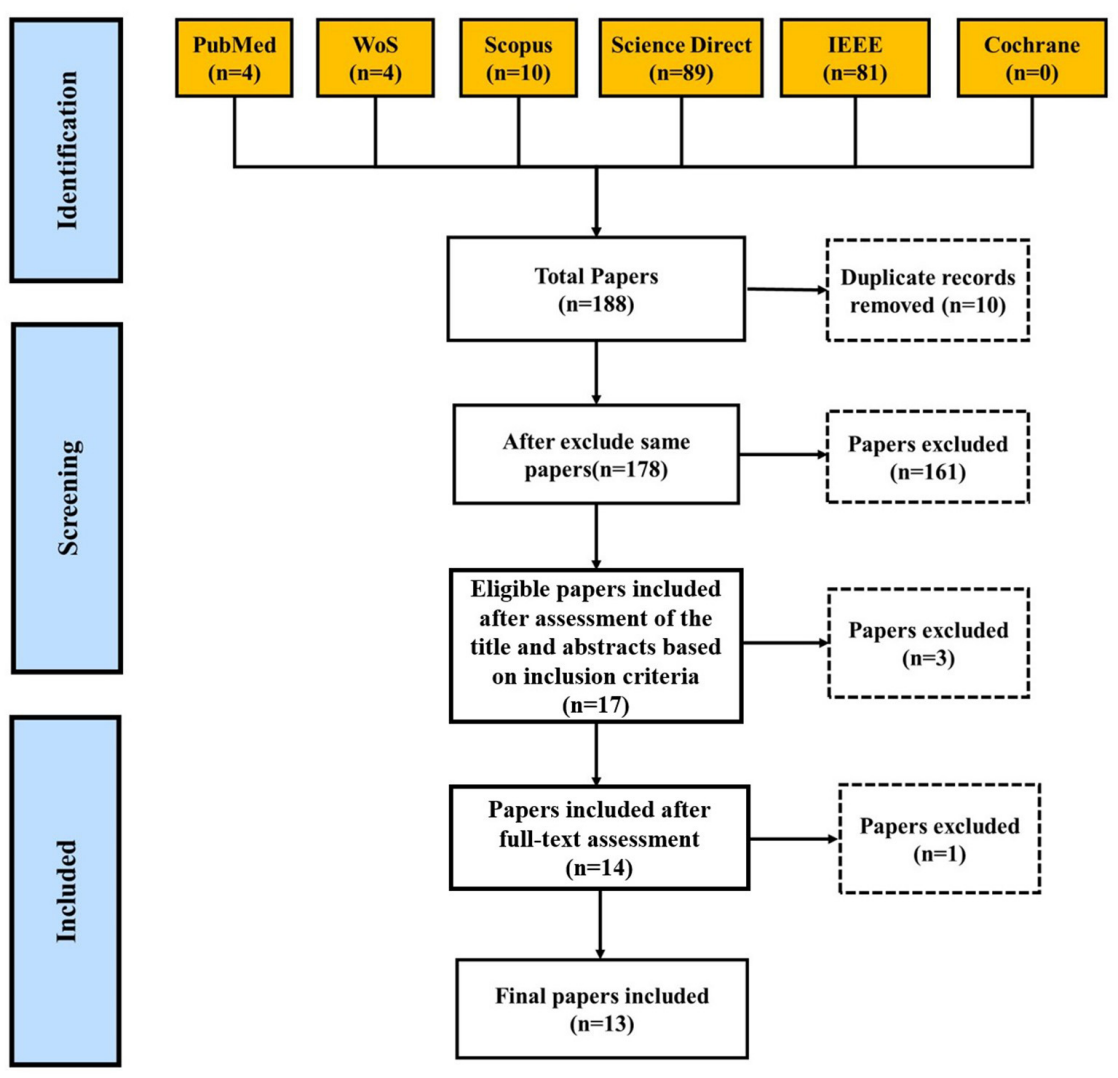
Scoping literature review procedure.

The combination of related keywords is demonstrated in Table 1. All the steps of searches were done based on the PRISMA checklist (Figure 2). The searches were independently done by three researchers to prevent possible bias. The search results referred to a third person who reviewed contradictions and made decisions in cases where there were disagreements. The searches were limited to papers published in the English without time limitation.

**Table 1:** Search strategy.

**Search strategy**	
**Databases**: Scopus, PubMed, Web of Science, IEEE, Science Direct and Cochrane	
**Limits**: Language (only resources with English), Species (studies on human)	
**Date**: 10 February to 10 March 2023	
**Search strategy**: #1 AND #2	
#1	“non-fungible token” OR “non-fungible token” OR “NFT”
#2	“health” OR “healthcare” OR “medical” OR “medicine”

### 3.4 Selection criteria

i. Only studies that explicitly mention the applications of NFTs are included.
ii. Only studies that show any use cases of NFTs in healthcare with health promotion goals included.
iii. Only original research included.
iv. Only English publications included.
v. Only journal articles and conference papers included.

### 3.5 Selection process

The process of article selection was based on PRISMA and 13 papers were selected (Figure 2). All the steps in the selection and evaluation of the quality of the papers were done by two researchers. Cases of disagreement were referred to a third person to make the final decision. We will also assess the quality and risk of bias of the included studies using established tools, such as the Cochrane Risk of Bias tool and the Newcastle-Ottawa Scale (46).

## 4 Results

According to predefined inclusion and exclusion criteria, the present study incorporated a total of 13 research investigations from an initial pool of 188 studies identified in the initial database search. The comprehensive delineation of these selected studies, including the various article categories, is displayed in Table 2. Table 2 shows the breakdown of article categories and presents information about the selected studies. The data extraction form had eleven categories, including the author's name and publication year, journal or conference name, country, type of study, methodology, blockchain platform, Technology Readiness Level (TRL), applications of NFTs in healthcare, study aims and main results, evaluation type and results and value proposition.

**Table 2:** Summary of the identified literature.

**Authors and year**	**Country**	**Journal name or congress name**	**Type of study**	**Methodology**	**Blockchain platform**	**Technology Readiness Level (TRL)**	**Applications in healthcare**	**The study aims and main results**	**Evaluation type and results**	**Value proposition**
Turki et al. (47))	Tunisia	Journal of King Saud University - computer and information sciences	Developmental research	SDLC	Ethereum blockchain based on solid contracts	TRL-7	Supply chain management in the pharmaceutical	They developed and evaluated an IoT and NFT-based approach for medicines lot Traceability in a Healthcare supply chain. By using NFT on Ethereum smart contracts, it appears an actor can easily trace and prove the existence and ownership of drug lots. Furthermore, in their system, some actors can earn royalties each time a successful trade occurs on any tokenized medicine lot.	They tested and validated the approach's effectiveness in enhancing drug traceability supply chains, as well as an analysis of the approach's costs and security.	Increased traceability and decreased costs
Shae and Tsai (48)	Taiwan	IEEE Third international conference on cognitive machine intelligence	Conceptual research	Reviewed and provided a blockchain model as a conceptual model	NA	TRL-3	Health data management	This article created a blockchain-based ecosystem platform to provide controlled and secure access to medical data. This blockchain platform unlocks the academic and business value of the medical data by modeling it as NFT, providing incentives for all participants along the data-driven value chain.	NA	Increased privacy and security
Musamih et al. (49)	United Arab Emirates	IEEE transactions on engineering management	Developmental research	SDLC	Ethereum blockchain based on solid contracts	TRL-7	Supply chain management in healthcare	They proposed a non-fungible-token-based solution for the management of healthcare products, where the ownership of a product is maintained by using digital certification, the trade and delivery of healthcare products is facilitated by a smart contract, and disputes are settled by an arbitrator while keeping all related information on-chain for auditing purposes. They utilized the Interplanetary File System to store the metadata of healthcare products to avoid storing large-sized data on the blockchain.	They conducted security testing to demonstrate that their solution was resilient and secure against common vulnerabilities and exploits. They compared their solution with existing solutions to show its distinctive features and novelty. They showed the novelty and unique features of their solution by comparing it with existing solutions. They illustrated how their solution could be generalized and extended to fit the needs of other applications.	Increased decentralization, transparency, security, reliability, auditability, and trust.
Mishra and Qi (50)	USA	Review of business	Conceptual research	-	NA	TRL-3	Drug discovery	In this paper, they proposed a peer-to-peer business model for drug discovery, which democratizes the drug discovery process and reduces drug prices by cutting the intermediaries that stand between biomedical researchers and future patients.	Using extensive simulations, they showed that in the NFT mega-fund, both senior and junior tranche investors had their principals fully repaid 99.9% of the time.	Increased repaid speed
Liu et al. (51)	China	International journal of environmental research and public health	Conceptual research	Mixed quantitative and qualitative methods	NA	TRL-3	Information modeling for healing and therapeutic design	This paper aimed to develop a conceptual Blockchain enhanced Information Modeling for Healing and Therapeutic Design framework that addressed the challenges in the HTD and promoted health and wellbeing. The structure of BC-HTD framework was two-fold: (1) a conceptual high-level framework comprising three levels: user; system; and information, (2) a conceptual low-level framework of detailed content at the system level. This paper analyzed the process of BC enhanced HTD and the knowledge management of HTD to aid design decisions in managing design information.	The conceptual BC-HTD framework was validated by industry experts and academics.	The form of NFT based on the blockchain facilitates mobilizing the healing and therapeutic behaviors of designers in the use of the advantage potential of healing and therapeutic design to promote health.
Kim et al. (52)	USA	2018 IEEE 9th annual information technology, electronics and mobile communication conference	Conceptual research	NA	Ethereum blockchain based on solid contracts	TRL-3	Supply chain management in food industry	This paper introduced Harvest Network, a theoretical end-to-end, vis à vis “farm-to-fork,” food traceability application integrating the Ethereum blockchain and IoT devices exchanging GS1 message standards. The goal was to create a distributed ledger accessible for all stakeholders in the supply chain. Their design effort created a basic framework (artifact) for building a prototype or simulation using existing technologies and protocols	NA	Increased transparency in supply chain management.
Jayasinghe et al. (47)	Sri Lanka	2022 13th international conference on computing communication and networking technologies	Developmental research	SDLC	Ethereum blockchain based on solid contracts	TRL-7	Health data management for EHR	This project aimed to protect Electronic Health Records by utilizing blockchain technology and NFT to ensure the confidentiality, integrity, and availability of patient data.	Security tests	Increased confidentiality, integrity, and availability of the EHR
Do Hoang et al. (53)	Vietnam	2022 RIVF international conference on computing and communication technologies (RIVF)	Developmental research	SDLC	Hyperledger Sawtooth vl.2.6	TRL-7	Health data management for EHR	This study aimed to improve the security and privacy of exchanging EHRs using blockchain technology, IPFS, and attribute-based encryption (ABE). The study used decentralized databases and a permissioned blockchain network with multiple nodes to ensure data availability and secure data in case of incidents. BABE Health is intended to improve healthcare management systems and deal with known security flaws.	To evaluate the performance of the proposed system, they considered the latency of transaction creation, the change in data size when applying encryption, and the encryption time with various data quantities.	Improved security and privacy of EHR
Do Hoang et al. (53)	USA	Cardiovascular digital health journal	Developmental research	The Solana Programming Language command-line interface (SPL CLI) was used to create a non-fungible token	Solana Blockchain with smart contracts	TRL-3	Health data management, NFT for EKG	The Solana Network created an NFT of an EKG as a proof-of-concept on the Solana blockchain, a third-generation cryptocurrency that is the third-largest in the world by market cap. The cost of the NFT was approximately $1.20 USD and could easily be automated. Web-hosting was required to house the media file, but decentralized storage using blockchain technology could be used to securely house the file. Changing EKGs to high-quality ASCII representations using algorithms created for that purpose would be an immediate way to store the data permanently on the blockchain itself.	NA	Improved security and accessibility
Do Hoang et al. (53)	USA	Cardiovascular digital health journal	Conceptual research	They propose a schema whereby de-identified EKG metadata stored on the public blockchain could be associated with a patient-specific identifier on a second layer accessible only to healthcare institutions and patients. This is accomplished utilizing a system of smart contracts and NFTs.	Solana blockchain	TRL-2	Health data management, NFT for EKG	The study identified a method that allows for the storage of healthcare data on the public blockchain while maintaining patient confidentiality. They researchers believed that globally accessible EKG data using public blockchain technology is achievable today. They planned to create an EKG storage protocol to define a system for its governance via a decentralized autonomous organization (DAO) and to address security concerns related to the use of a second layer.	NA	Maintaining patient confidentiality
Gebreab et al. (54)	United Arab Emirates	IEEE access	Developmental research	SDLC	Ethereum blockchain based on solid contracts	TRL-8	Supply chain management in medical devices	They proposed a non-fungible token (NFT) based on a solution that exploited blockchain smart contracts, integrated tokenization protocols, and utilized a decentralized storage system for a reliable and efficient medical devices traceability system and ownership management solution.	The paper also presented testing details and analyses of the solution's feasibility through a cost and security analysis.	Increased security and reliability
								In their proposed system, NFTs are used to represent the digital twin of the medical device. This digital twin captures the medical device attributes and its relevant metadata during its life cycle from production, manufacturing, distribution and movement to current use and ownership. They present the system architecture and implementation details, along with tested algorithms that define the various functions of our smart contracts.		
Gebreab et al. (54)	Italy	Computer communications	Developmental research	SDLC	NA	TRL-6	Infection tracking and notification system	This paper presented a contact tracing protocol based on blockchain technology that exploits smart contracts for reporting contacts at risk of contagion. The novelty of the proposed solution is the use of NFTs to guarantee user privacy through a decentralized approach, equipped with a reliable non-proprietary notification mechanism that allows public access to anonymous infections data. The proposed solution guarantees the privacy of system users and is equipped with a notification mechanism based on ERC721 tokens. The non-fungible nature of these tokens guarantees the impossibility of producing false notifications and guarantees the unrepeatable outcome of the diagnostic test.	To evaluate the performance of the proposed system, the Besu client configuration was measured. Also, contact tracing systems were compared, to provide more information about the performance of the proposed approach.	Increased user privacy
Bala et al. (55)	India	2022 IEEE international conference on blockchain and distributed systems security (ICBDS)	Developmental research	SDLC	Ethereum blockchain based on solid contracts	TRL-5	Health data management, NFT for	The authors presented a solution to the problems faced by the healthcare industry in maintaining electronic health records (EHR) and the supply chain for tracking medicines and identifying dishonest distributers. The insurance claim feature ensures that the money is transferred securely and handles all edge cases. The user-friendly user interface makes the whole process easy and quick for the client. The present problems can not only be solved with an Ethereum blockchain, but in future they can also work in wider scope in future, as mentioned above.	Performance evaluation	Increase security

Subsequent to data extraction, the study's outcomes were summarized and depicted in the form of tables and figures, aligning the presentation of results with the overarching study objectives. Also, to enhance clarity and facilitate comprehension, the results have been thoughtfully segregated for presentation, focusing on the primary columns of Table 2. This deliberate structuring offers a more organized and intuitive presentation of the research findings for the reader's benefit.

### 4.1 Literature distribution

**Figure 3:**
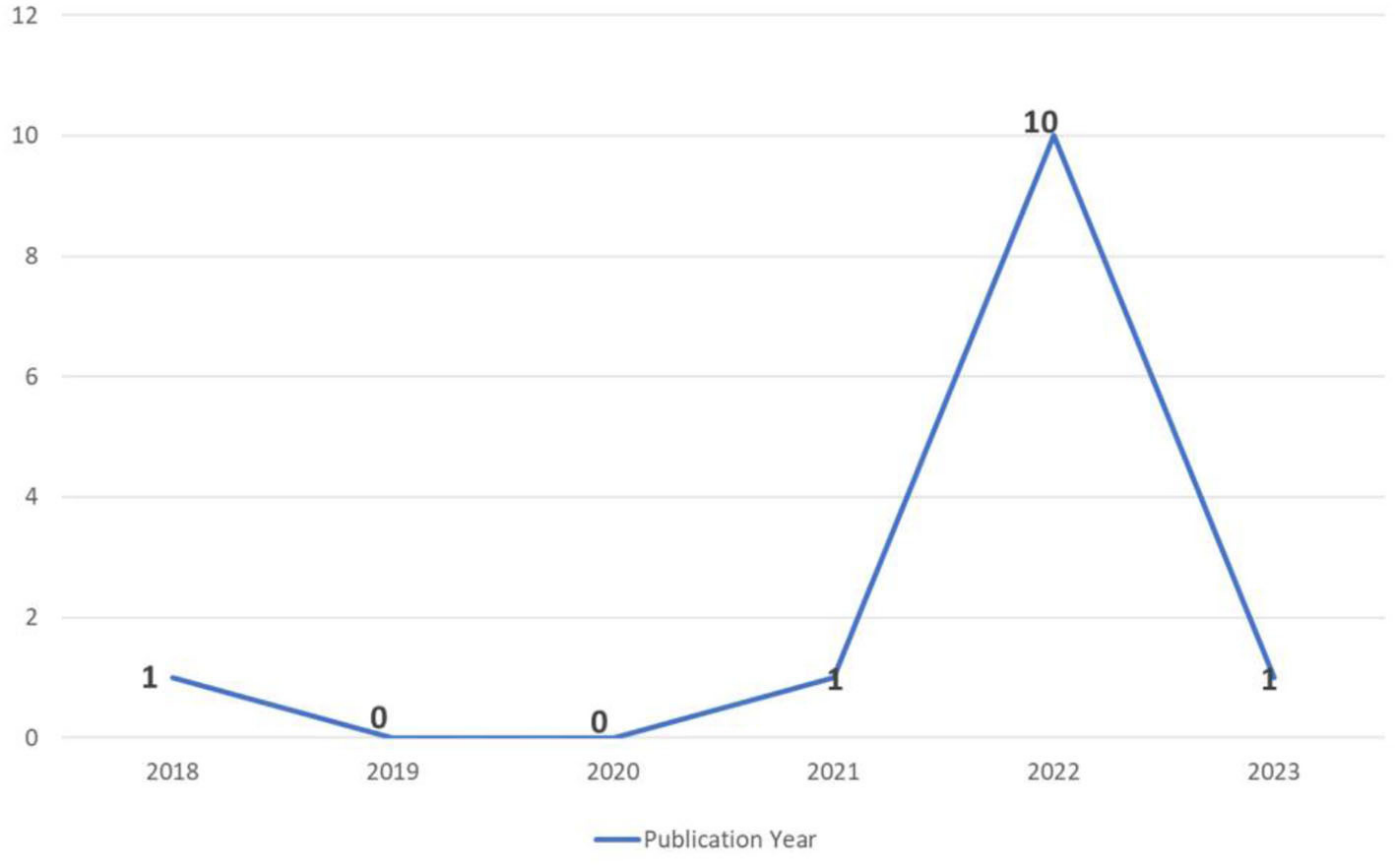
The number of publications published year since NFTs concept provided.

Most of the papers (77%) were published in 2022 (Figure 3). 62% of studies were published in journals while 38% were published as conference research. Furthermore, as mentioned in Table 3, a greater number of the publications were from USA (23%) and UAE (14%).

**Table 3:** Author frequency by nation.

**Countries**	**Frequency**	**Percent**
Tunisia	1	7.6%
Taiwan	1	7.6%
UAE	2	15.3%
USA	4	30.7%
China	1	7.6%
Sri Lanka	1	7.6%
Vietnam	1	7.6%
Italy	1	7.6%
India	1	7.6%

### 4.2 Type of study

**Figure 4:**
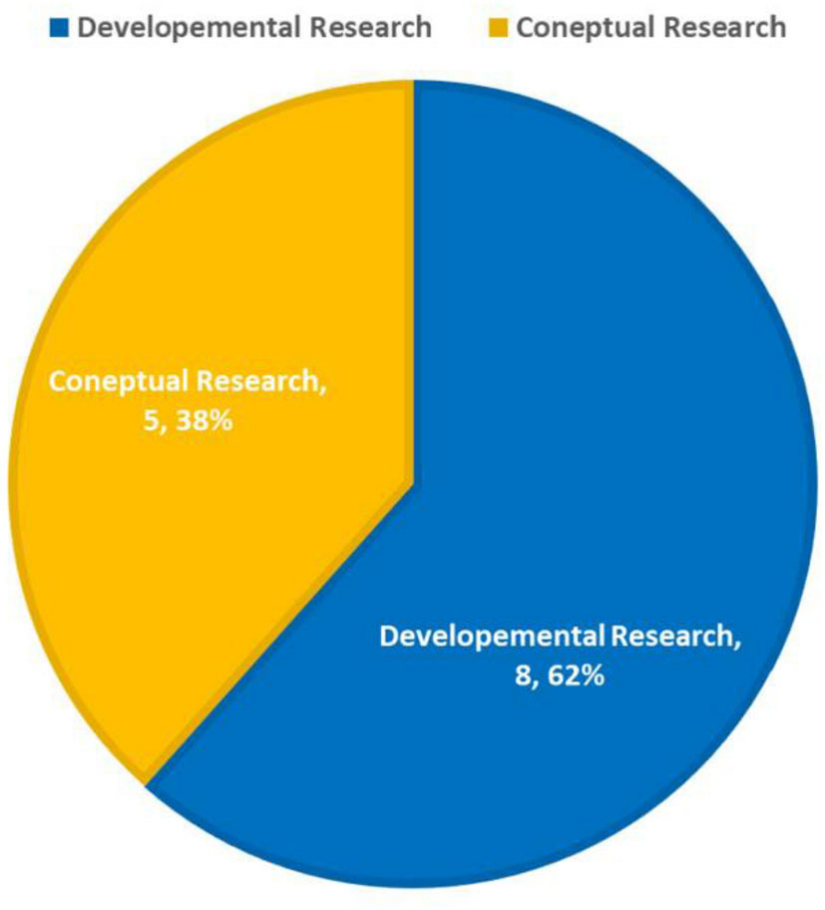
The frequency of type of study.

As presented in the fourth column of Table 2, the frequency (%) of type of study is presented in Figure 4.

### 4.3 Methodology applied in research

As showed in the fifth column of Table 2, the main methodology that applied in research is system development life cycle (SDLC) with main analysis, design, implementation and evaluation phases (54%).

### 4.4 Blockchain platforms applied in research

**Figure 5:**
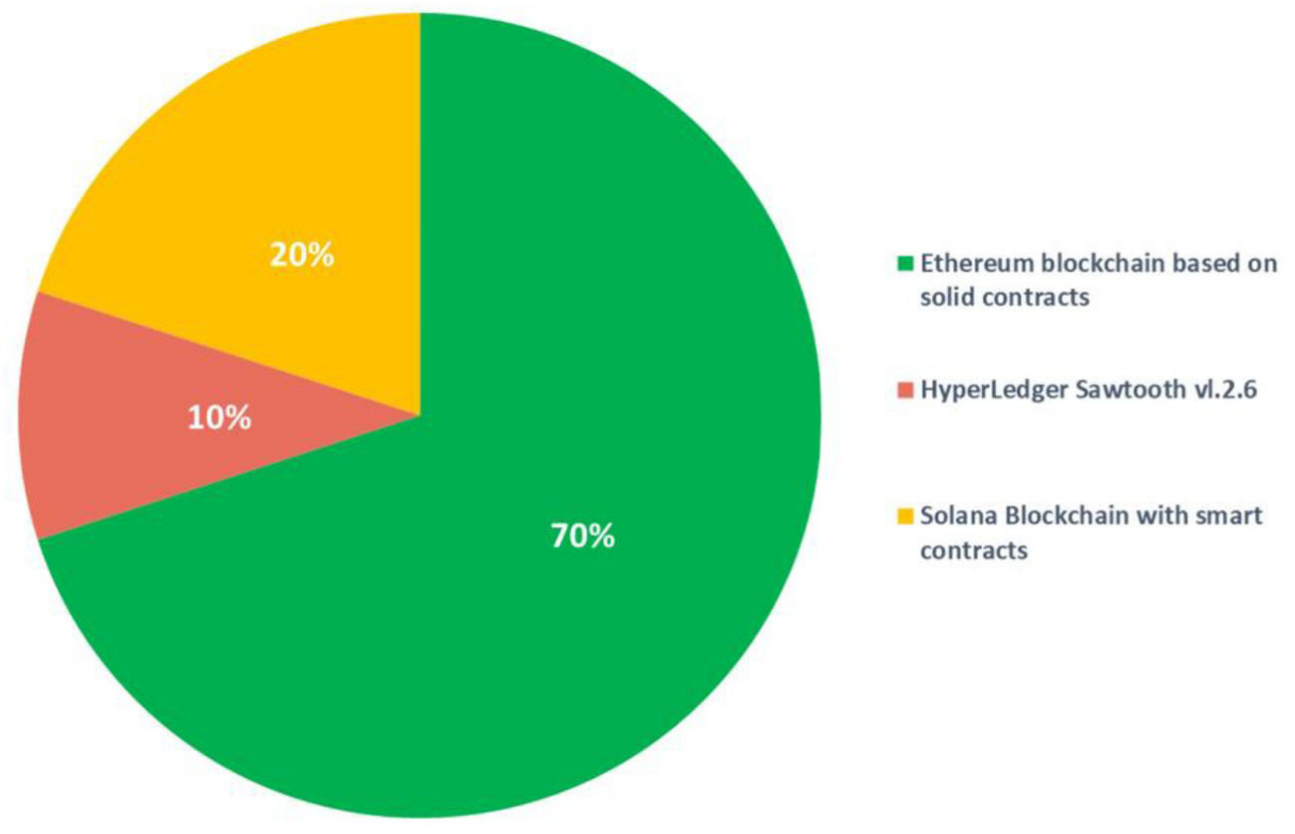
The frequency of types of blockchain platforms.

As presented in the sixth column of Table 2, the types of blockchain platforms applied in the research studied. The frequency (%) of types of blockchain platforms is presented in Figure 5.

### 4.5 Technology readiness level

**Figure 6:**
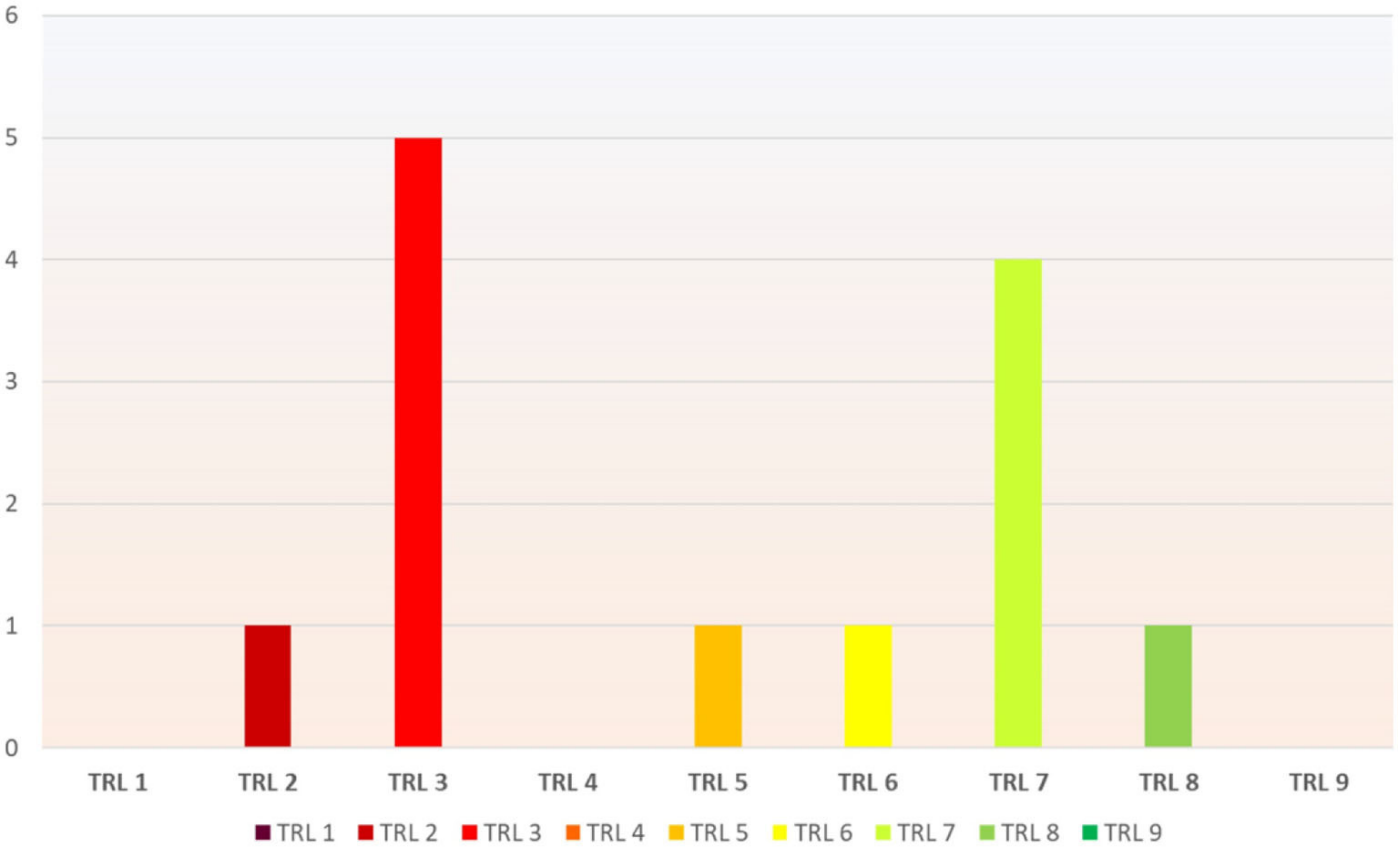
Distribution of NFTs-based solutions on the TRL model.

The seventh column of Table 2 illustrates the condition of the NFTs-based solutions on the TRL model. Figure 6 shows the number of research projects exist at each level. The TRL model assesses the development stage of technologies, ranging from concept (TRL 1) to mature deployment (TRL 9). It aids in decision-making for research, development, and innovation by providing a standardized framework to gauge technology readiness.

### 4.6 The applications of NFTs in healthcare

**Figure 7:**
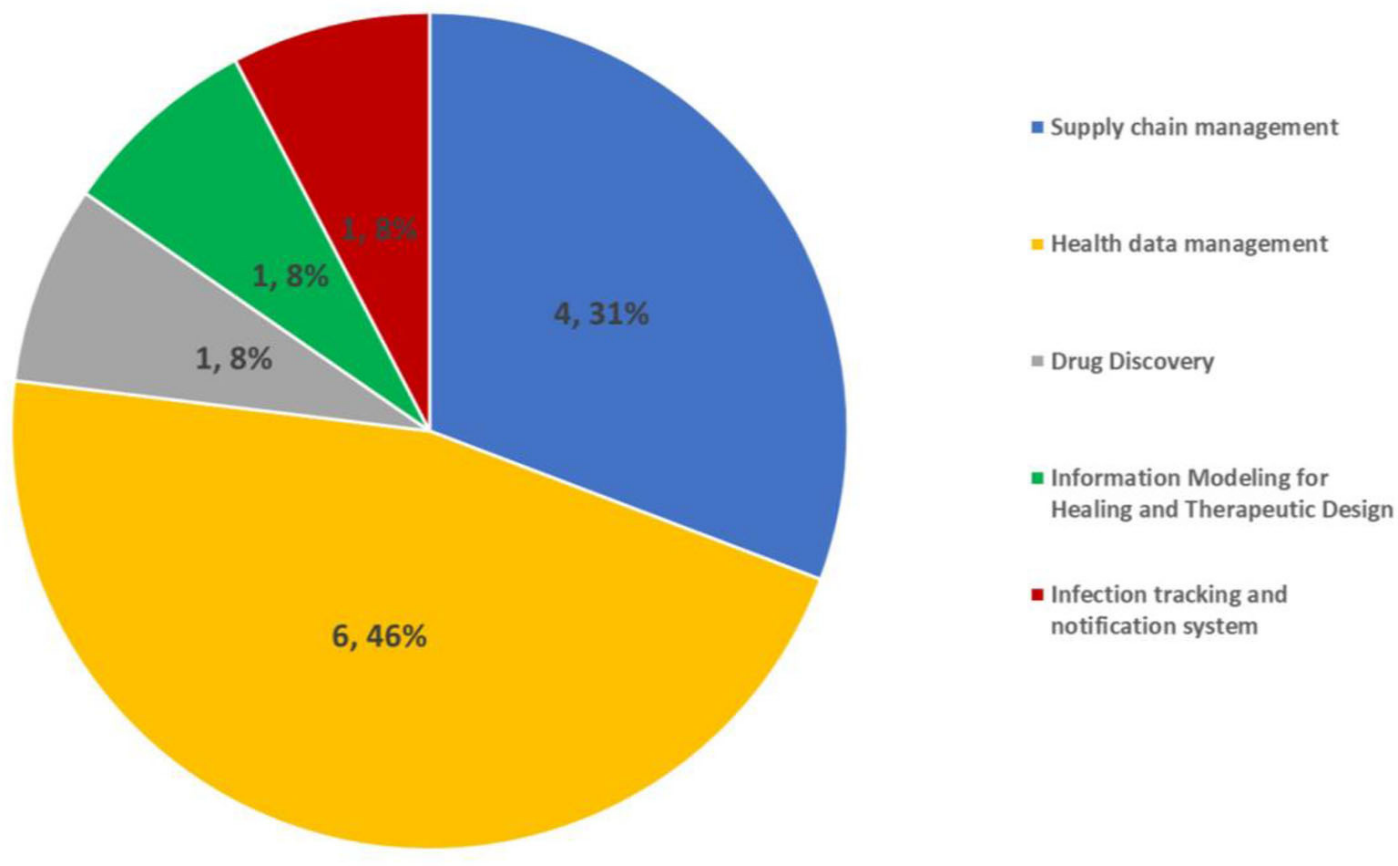
The frequency of applications of NFTs in healthcare.

As shown in the eighth column of Table 2, the applications of NFTs in healthcare were studied. Figure 7 shows the applications of NFTs in healthcare by proportion. The results revealed that the most used health application of NFTs was for health data management at 46%. Next, the second most frequently used application of NFTs in healthcare was for supply chain management at (31%).

### 4.7 Evaluation type and results

As presented in the tenth column of Table 2, the evaluation type and results were the primary variables investigated. About 69% of studies reported information related to the evaluation phase. Of the 9 studies that reported information on the evaluation phase, most evaluation methods focused on security evaluation, comprising five studies or 55%.

### 4.8 Value proposition

The eleventh column of Table 2 illustrates the value proposition of NFTs base solutions. The results showed the most frequent value proposition of these solutions were increasing security, privacy, confidentiality, transparency, and accessibility.

## 5 Discussion

NFTs have gained a lot of attention in recent years as a means of establishing ownership and authenticity of digital assets (13, 14, 19, 21, 23). In the context of healthcare, NFTs have the potential to be used in a variety of ways, thus, this study set out to determine the applications of NFTs in healthcare (18, 42, 43, 49). In fact, the study goals included exploring the possibilities of NFTs in healthcare, including their potential to improve the transparency and security of healthcare data, streamline the supply chain for medical products, and facilitate the exchange of medical information between healthcare providers. By understanding the applications of NFTs in healthcare, we can explore ways to improve the efficiency and effectiveness of healthcare delivery.

### 5.1 Literature distribution

The findings of this study indicate a significant increase in the utilization of NFT-based solutions within the healthcare industry over the past 2 years, particularly in 2022. Specifically, the investigation revealed that 10 out of the 13 studies included in the analysis were conducted in 2022. These results align with other studies that have identified a growing trend in publications that focus on the applications of NFTs worldwide (22, 33). Collectively, these observations underscore the importance of NFTs for future research and development. Additionally, this study identified the USA and UAE as the most active countries in terms of conducting original research in this domain, accounting for 46% of the studies included in our analysis. These findings are consistent with previous research done in this area (13, 22, 27, 28).

### 5.2 Type of study

Another noteworthy finding is that 62% of the studies conducted in this domain were classified as developmental studies, with the remaining 38% falling into the category of conceptual research. An important related fact is that the applications of NFTs in healthcare extend beyond the realm of theoretical and conceptual frameworks, with researchers actively working to implement these solutions in developmental and practical contexts within healthcare. This observation is consistent with our earlier findings, which indicated that most investigations focused on the applications of NFTs, in general, were not restricted to conceptual frameworks alone (22).

### 5.3 Applied methodology

Given that most of the studies included in this analysis were classified as developmental, it is noteworthy that the system development life cycle emerged as the primary applied methodology across these investigations. SDLC is a methodology used in software engineering to guide the development of information systems, software applications, and other technology-based projects. It provides a framework for the development of software and systems from inception to retirement, with the aim of producing high-quality, efficient, and effective solutions that meet the needs of stakeholders (56).

### 5.4 Blockchain platforms applied in research

Our analysis reveals that approximately 70% of the researched or proposed projects in this study utilized the Ethereum blockchain, while 20% employed Solana, and 10% chose Hyperledger Sawtooth. Notably, Ethereum stands as the dominant choice for NFT applications in healthcare due to factors such as its robust developer community, market value, inherent support for Decentralized Applications (DApps), tokenization capabilities (i.e., creating new coins on the Ethereum network), and backing for non-fungible digital assets. It's important to note that while Ethereum typically follows the ERC-20 standard (57), which produces fungible tokens, NFTs on the Ethereum network deviate from this standard, making them unique and non-interchangeable. Although this deviation may be resolved in the future, it raises concerns in the healthcare sector where security, ethical, and compliance standards are exceptionally high, warranting careful consideration of NFT non-compliance (17, 24, 58–60). Support for smart contracts is not an exclusive Ethereum technology, as Hyperledger and Solana both support this feature.

Aside from the mapped blockchain solutions capabilities, limitations that were not addressed or needed to be better addressed for the successful application of NFTs for healthcare are presented in Table 4 (28, 31, 34, 61). It is important to understand that some features may be limitations but only in certain use cases. Cells marked with an “X” mean that the limitation is known by the blockchain's community.

**Table 4:** Comparison of limitations of blockchain platforms used in NFTs in healthcare.

**Limitation**	**Ethereum**	**Solana**	**Hyperledger sawtooth**
Scalability	X		
Limited transaction throughput	X		X
Energy consumption	X	X	
Standardization	X	X	X
Centralization	X	X	X
Decentralization^*^	X	X	
Development tools		X	X
Complexity			X
Limited smart contract support			X
Interoperability	X	X	X
Storage costs	X	X	
Transaction speeds	X		
Security	X	X	
Limited community support		X	X

Although many of the limitations listed above are equally relevant, interoperability, standardization, and centralization were found to be of high importance across all technologies for the healthcare domain. For example, centralization regards imbalances in consensus algorithms that would impact NFTs minting and sales, impacting data owners. Standardization regards the implementation of technologies, standards, frameworks, and policies agreed upon by a committee. If standardization is not present at some level to regulate how NFT are created, the risk for users is increased (62). Finally, interoperability impedes the interchange of NFTs between one blockchain architecture and another, limiting users' data portability, which is highly important in the health domain (28, 31). Hence, excluding all cybersecurity aspects since they were out of the scope of this work, these are the main limitations that NFTs technologies must overcome before being deemed ready to manage healthcare and personal data from patients reliably and competitively.

### 5.5 Technology readiness level

Based on the Technology Readiness Level (TRL) scale (63), the present investigation revealed that approximately half of NFT-based solutions were in the early stages of research and conceptualization, while the remaining half had progressed to the developmental and operational levels. These findings suggest that the concept of NFTs is relatively nascent, and research aimed at developing NFT-based solutions is ongoing. Nevertheless, the results indicated that NFT-based solutions in healthcare hold significant promise for addressing critical healthcare challenges and can be implemented effectively in real-world settings.

### 5.6 The applications of NFTs in healthcare

**Figure 8:**
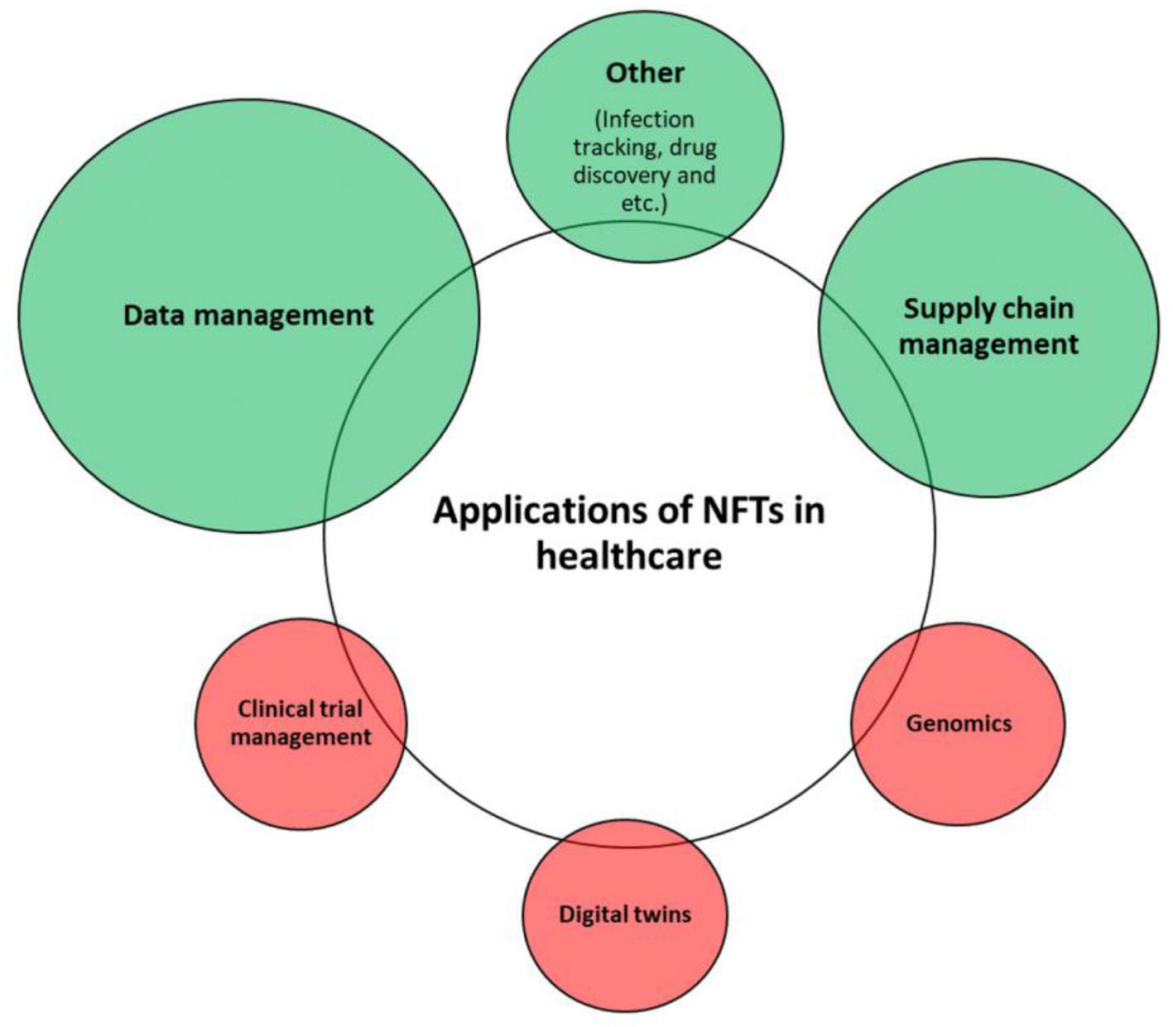
The current state of applications of NFTs in healthcare.

The classification of NFTs applications in healthcare utilized in this study was primarily based on the work of Musamih et al. (44). Based on this classification, the most significant applications of NFTs in healthcare were grouped into five distinct categories, including: (1) supply chain management, (2) patient-centric data management, (3) digital twins, (4) clinical trial management, and (5) genomics. The most important finding from the literature was that the majority of NFTs use cases in healthcare pertain to data management and supply chain management (44). However, the present study did not identify any instances classified under the remaining categories of digital twins, clinical trial management, and genomics. However, it is important to note that the present study does not refute the findings of Musamih et al.'s (44) research Given that NFTs are still a relatively novel concept, it is plausible that many of the potential applications of NFTs in healthcare discussed by Musamih et al. (44) have yet to be explored by scholars and may be leveraged in the future. This study has also revealed additional applications of NFTs in healthcare. Specifically, three studies proposed NFT-based solutions for drug discovery, infection tracking, and utilizing information modeling for designing healing, and therapeutic interventions. Therefore, as shown in Figure 8, the most important current and future applications of NFTs in healthcare can be considered as follows (in Figure 8, circle sizes represent the frequency of NFT applications in the healthcare sector, as determined from our review results. Green circles signify NFT applications identified in our review, while red circles denote NFT applications mentioned in other studies).

#### 5.6.1 Health data management

NFTs can offer several benefits for health data management, such as enhancing the security and privacy of sensitive patient information through encryption and decentralized storage on a blockchain network. NFTs can also enable seamless and tamper-proof tracking of medical records, facilitating more accurate diagnoses and treatments. Moreover, NFTs can provide patients with greater control over their health data by empowering them to grant permission for access to their records and receive compensation for its use (16, 42, 44, 47, 48, 53, 64, 65).

#### 5.6.2 Supply chain management

NFTs can be utilized in healthcare supply chain management to improve transparency, traceability, and accountability. By creating a secure and tamper-proof record of the transportation of medical supplies and equipment, NFTs can help to prevent counterfeiting and improve efficiency in the distribution process. Additionally, NFTs can enable better tracking of the temperature and conditions in which medical products are stored and transported by attaching a unique digital certificate to each item that can be tracked on the blockchain to ensuring that they remain viable and safe for use (42, 49, 52, 54, 66).

#### 5.6.3 Genomics

NFTs can have potential applications in genomics by providing secure and efficient management of genetic data. By creating a tamper-proof record of genomic information, NFTs can enable more accurate and personalized healthcare interventions. NFTs can also facilitate secure sharing and storage of genetic data for research purposes while preserving patient privacy. Furthermore, NFTs can allow for equitable and transparent distribution of financial benefits arising from the use of genetic data (44).

#### 5.6.4 Digital twins

NFTs can be applied to create digital twins, which are virtual models of patients that can be used to simulate medical scenarios and optimize treatments. By creating a tamper-proof record of a patient's health data, NFTs can enable more accurate and personalized digital twin models. Additionally, NFTs can allow for secure and transparent sharing of digital twin data for research purposes while protecting patient privacy. Furthermore, NFTs can enable patients to have greater control over the use of their digital twin data and receive compensation for its use (44).

#### 5.6.5 Other

##### 5.6.5.1 Infection tracking

NFTs can safeguard the tracking of the spread of infectious diseases by creating tamper-proof records of patient health data and facilitating secure sharing of information between healthcare providers. Generally, NFTs can help to improve infection tracking in healthcare by creating a tamper-proof record of the movement of people and equipment within healthcare facilities. By attaching NFTs to equipment or even patient wristbands, healthcare providers can track the movement of people and things within their facilities, making it easier to trace the spread of infections. NFTs can also be used to record the temperatures of equipment used in patient care, such as ventilators or IV pumps, ensuring that they are within safe operating ranges. This information can be used to quickly identify potential sources of infection and promote more effective disease control measures (67).

##### 5.6.5.2 Pharmaceutical research and drug discovery

NFTs can potentially accelerate the drug discovery process by enabling secure and transparent sharing of research data and intellectual property among researchers and organizations. By creating an immutable record of ownership and usage rights, NFTs can facilitate collaborations while also protecting the intellectual property of researchers. This can lead to more efficient drug discovery and development processes, ultimately leading to the creation of new treatments for a variety of health conditions. Additionally, NFTs can be used to tokenize the data generated during clinical research, making it easier for researchers to analyze and share data, leading to faster and more accurate drug development (40).

##### 5.6.5.3 Information modeling for designing healing and therapeutic interventions

NFTs can be used to create information models for designing healing and therapeutic interventions, allowing for the development of more personalized and effective treatment plans. By creating digital representations of patient health data, NFTs can enable the analysis of large amounts of information and support data-driven decision making for healthcare providers (51).

According to the findings of this study, the green circles represent current applications of NFTs in healthcare, while the red circles denote potential future use cases of NFTs within the healthcare industry.

### 5.7 Evaluation type and results

Another noteworthy discovery from this investigation is that most studies included in the analysis incorporated evaluation phases. This finding is significant in that it highlights the importance of assessing the performance of NFT-based solutions and draws attention from researchers and stakeholders in this domain. Additionally, the results of this study revealed that the primary dimension evaluated in these investigations was security. One potential explanation for this phenomenon may be attributed to the significant advantage of utilizing NFT-based solutions, which is the enhancement of security. This finding is consistent with other investigations (31).

### 5.8 Value proposition

The present study identified the main value propositions of NFT-based solutions in healthcare which primarily centered around enhancing security, privacy, confidentiality, transparency, and accessibility. The present study aligns with previous research that demonstrates the significant benefits of NFTs-based solutions, including unique digital ownership, enhanced security and privacy, the potential for increased value, traceability, accessibility, and interoperability (22, 27, 31).

While the potential applications of NFTs in healthcare such as improve data security, track the provenance of pharmaceuticals and medical devices, and streamline the supply chain for medical products are promising, however, it is important to acknowledge and address the associated challenges and concerns. First and foremost, the integration of NFTs in healthcare systems may introduce security vulnerabilities, as healthcare data is highly sensitive and susceptible to cyberattacks (24, 44, 49, 68). Ensuring robust security measures and compliance with healthcare data regulations, such as HIPAA in the United States, is imperative. Moreover, the high energy consumption associated with blockchain technologies, especially Ethereum, which is a prominent platform for NFTs, raises concerns about environmental sustainability. The carbon footprint of blockchain operations must be considered and mitigated to align with healthcare's commitment to ecological responsibility. Additionally, the storage requirements for NFTs, which often involve large digital files, may pose a significant challenge in healthcare settings where data storage and management can be resource-intensive. This issue may require substantial infrastructure investments to ensure the secure and efficient storage of NFT-related healthcare data (24). Furthermore, the cost associated with deploying and maintaining NFT-based solutions in healthcare can be substantial, potentially limiting their widespread adoption, especially in resource-constrained healthcare systems (24, 44, 49, 69). The financial implications of implementing and managing NFTs need to be carefully evaluated to assess their feasibility and sustainability. Another limitation is the potential for over-reliance on NFTs, which may lead to exclusion or discrimination of individuals who lack access to this technology, potentially exacerbating healthcare disparities. Furthermore, the legal and ethical aspects of ownership, licensing, and consent for medical data represented by NFTs need careful consideration, as they might raise complex issues regarding patient rights, informed consent, and intellectual property (44, 49, 69, 70). Additionally, the adoption of NFTs in healthcare faces challenges in gaining acceptance by health policy makers and healthcare providers, who may be hesitant to embrace this novel technology. Lastly, the nascent stage of NFT adoption in healthcare implies that the scalability and interoperability of NFT-based solutions are yet to be fully tested. As the technology matures, these challenges and limitations should be taken into account to ensure that NFTs truly deliver their potential benefits to the healthcare sector (42, 44, 70). By addressing these limitations and concerns, NFTs have the potential to improve healthcare delivery and patient outcomes.

Also, it is essential to differentiate between the applications of NFTs and the broader blockchain technology in the context of healthcare due to their unique contributions to the industry. While both NFTs and blockchain offer secure and transparent data management, NFTs stand out for their distinct ability to represent ownership, authenticity, and provenance of digital assets. This differentiation becomes crucial when addressing healthcare challenges, as certain applications may require the specific features that NFTs provide. By understanding when and where NFTs are most advantageous, healthcare stakeholders can make informed decisions about technology adoption, potentially leading to more effective, efficient, and patient-centric solutions. Moreover, a clear distinction between NFTs and blockchain allows for a more precise analysis of how each technology contributes to resolving healthcare issues, ensuring that the appropriate tools are applied to the task at hand. In our ongoing research, we aimed to elucidate the precise roles of NFTs and broader blockchain technology in healthcare applications, shedding light on the distinct advantages and synergies that drive innovation in the field (34, 44, 49, 61).

While this scoping review provides valuable insights into the burgeoning utilization of NFTs in healthcare, it is essential to acknowledge certain limitations in our study. Firstly, the relatively limited number of selected papers (13) may not encompass the entirety of NFTs applications in healthcare, and there might exist additional relevant research that was not included. Furthermore, the rapidly evolving nature of both blockchain and NFTs technologies implies that the field is subject to continuous developments and advancements that may not be fully captured in our review. Additionally, the potential biases inherent in the selection and analysis of the included studies, as well as the use of predetermined search criteria, could influence the comprehensiveness of the findings. Therefore, while our review underscores the transformative potential of NFTs in healthcare, it is important to consider these limitations as areas for further research and exploration in this dynamic and innovative domain.

Future research in NFTs-based solutions for healthcare should focus on several areas to address gaps in the existing literature. First, more studies are needed to explore the ethical, social, and cultural implications of using NFTs in healthcare. This includes examining potential issues related to data privacy, ownership, and accessibility, as well as the impact on healthcare providers and patients. Second, future research should investigate how NFTs can be integrated into existing health systems and workflows, including exploring potential barriers to adoption and identifying strategies for effective implementation. Third, there is a need for more empirical studies to evaluate the effectiveness and efficiency of NFTs-based solutions in healthcare. Finally, researchers should explore the potential for using NFTs in other areas of healthcare beyond those currently identified. By addressing these areas of research, we can better understand the potential benefits and challenges of using NFTs in different areas of healthcare and develop strategies to maximize their value for patients, providers, and other stakeholders in the healthcare ecosystem.

## 6 Conclusion

This scoping review highlights the emerging use of NFTs in healthcare. The study searched for and reviewed 13 relevant papers, and the results provide valuable insights into the current state of NFTs in healthcare. The study also explored various variables, including type of study, methodology, technical aspects of NFTs-based solutions, TRL levels, applications of NFTs in healthcare, evaluation type and results, and the value proposition of NFTs. The results revealed that NFTs have potential applications in healthcare supply chain management, data management, clinical trial management, digital twin, genomic, and other areas. In addition, this study has uncovered further potential uses for NFTs in healthcare. More specifically, three of the reviewed studies proposed NFT-based solutions for drug discovery, tracking infections, and utilizing information modeling to design healing and therapeutic interventions. Additionally, increased security, privacy, confidentiality, transparency, and accessibility were found to be the most common value propositions of NFTs-based solutions. Overall, the results of this scoping review suggest that NFTs have the potential to revolutionize the healthcare industry; however, further research is needed to explore their full potential.

## Data availability statement

The raw data supporting the conclusions of this article will be made available by the authors, without undue reservation.

## Author contributions

SA: Conceptualization, Data curation, Formal analysis, Investigation, Methodology, Validation, Writing—original draft. PMo: Conceptualization, Project administration, Supervision, Writing—review & editing. PMi: Conceptualization, Data curation, Formal analysis, Validation, Writing—original draft. AG: Data curation, Formal analysis, Validation, Writing—original draft. TH-P: Conceptualization, Writing—review & editing. DC: Funding acquisition, Resources, Writing—review & editing.
